# Shift Work, Circadian Disruption, and Immune Dysregulation: Molecular Links to Gastrointestinal Diseases and Occupational Health Implications

**DOI:** 10.3390/diagnostics16183010

**Published:** 2026-09-17

**Authors:** Ancuța-Ramona Boicea Camen, Daniel Cosmin Caragea, Mihail Virgil Boldeanu, Dan Nicolae Florescu, Mohamed-Zakaria Assani, Isabela Siloși, Lidia Boldeanu

**Affiliations:** 1 Department of Occupational Medicine, University of Medicine and Pharmacy of Craiova, 200349 Craiova, Romania; ancuta.boicea@umfcv.ro; 2 Department of Nephrology, Faculty of Medicine, University of Medicine and Pharmacy of Craiova, 200349 Craiova, Romania; daniel.caragea@umfcv.ro; 3 Department of Immunology, Faculty of Medicine, University of Medicine and Pharmacy of Craiova, 200349 Craiova, Romania; mihail.boldeanu@umfcv.ro (M.V.B.); isabela.silosi@umfcv.ro (I.S.); 4 Department of Gastroenterology, Faculty of Medicine, University of Medicine and Pharmacy of Craiova, 200349 Craiova, Romania; 5 Research Center of Gastroenterology and Hepatology, University of Medicine and Pharmacy of Craiova, 200638 Craiova, Romania; 6 Doctoral School, University of Medicine and Pharmacy of Craiova, 200349 Craiova, Romania; 7 Department of Microbiology, Faculty of Medicine, University of Medicine and Pharmacy of Craiova, 200349 Craiova, Romania; lidia.boldeanu@umfcv.ro

**Keywords:** shift work, circadian disruption, gastrointestinal disorders, immune dysregulation, intestinal barrier, gut microbiota, chrononutrition, biomarkers, occupational medicine, precision medicine

## Abstract

Shift work is an essential component of modern occupational systems but represents a major source of circadian misalignment associated with gastrointestinal symptoms and selected gastrointestinal disorders. The biological pathways underlying these associations remain incompletely integrated across circadian, neuroendocrine, immune, epithelial, and microbial domains. This narrative review aimed to synthesize current evidence linking shift work with gastrointestinal dysfunction and disease while distinguishing epidemiological associations from experimental mechanistic evidence and biologically plausible but insufficiently validated relationships. We conducted a structured literature search in PubMed/MEDLINE, Scopus, and Web of Science, focusing primarily on studies published between January 2020 and July 2026. We prioritized recent original studies, systematic reviews, meta-analyses, and mechanistic and translational investigations, while retaining relevant landmark studies. Evidence was organized within an integrative framework encompassing occupational exposure, circadian disruption, neuroendocrine alterations, immune dysregulation, intestinal barrier dysfunction, gut microbial and metabolic alterations, and gastrointestinal outcomes, with explicit consideration of differences in evidentiary strength across these proposed relationships. Human studies consistently support an association between night or rotating shift work and circadian disruption, whereas evidence for downstream neuroendocrine, immune, epithelial, and microbial pathways varies substantially in directness and strength. Experimental studies indicate that circadian disruption can alter inflammatory signaling, epithelial barrier function, and host–microbiota interactions, while microbial metabolites, including short-chain fatty acids, secondary bile acids, and tryptophan derivatives, may participate in reciprocal interactions with mucosal immunity and barrier integrity. Epidemiological evidence is strongest for disorders of gut–brain interaction, particularly irritable bowel syndrome, whereas evidence for functional dyspepsia, inflammatory bowel disease, and colorectal neoplasia is more limited, inconsistent, or unresolved. Candidate circadian, inflammatory, intestinal barrier, microbial, and metabolomic biomarkers remain investigational and are not currently validated for gastrointestinal risk prediction or routine occupational surveillance. Shift work is associated with selected gastrointestinal outcomes, while experimental evidence provides biological plausibility for interconnected circadian, neuroendocrine, immune, epithelial, and microbial mechanisms. However, the proposed framework should be regarded as integrative and hypothesis-generating rather than as an established linear causal cascade in human shift workers. Prospective longitudinal and interventional studies are required to establish temporal and causal relationships, validate candidate biomarkers, and determine whether occupational, behavioral, or mechanism-based interventions can produce clinically meaningful gastrointestinal benefits.

## 1. Introduction

Shift work has become an indispensable part of modern society, ensuring the continuous operation of healthcare systems, manufacturing industries, transportation, emergency services, public safety, and numerous other sectors that require uninterrupted activity. Approximately one-fifth of the workforce in industrialized countries works non-standard schedules, including permanent night shifts, rotating shifts, evening shifts, and irregular work patterns. Rather than representing merely an alternative work schedule, shift work constitutes a multidimensional occupational exposure characterized by varying combinations of nocturnal light exposure, sleep restriction and fragmentation, irregular meal timing, psychosocial stress, prolonged wakefulness, and disruption of normal work–rest cycles. These exposures are strongly associated with circadian misalignment and may affect neuroendocrine, metabolic, cardiovascular, immune, and gastrointestinal physiology [1,2,3,4].

Among the diverse health consequences of shift work, gastrointestinal disorders have emerged as one of the most prevalent yet frequently underestimated occupational health problems. Epidemiological studies have reported higher rates of gastrointestinal symptoms and disorders among rotating and night-shift workers than among fixed-day workers. A systematic review and meta-analysis by Chang et al. found a 56% higher overall risk of gastrointestinal disorders among rotating shift workers compared with fixed-day workers, with particularly strong associations for indigestion and peptic ulcer disease (PUD) [3]. Meta-analytic evidence also indicates an increased prevalence of irritable bowel syndrome (IBS) among shift workers, whereas the association with functional dyspepsia has been less consistent [5]. More recently, large prospective data have demonstrated an association between night-shift work and incident IBS, including exposure–response relationships with the duration and frequency of night-shift work [6]. Importantly, however, the epidemiological evidence is not equivalent across gastrointestinal outcomes, and associations with inflammatory bowel disease (IBD), colorectal cancer (CRC), and other gastrointestinal diseases remain less consistent or insufficiently established.

Despite growing epidemiological evidence, the biological mechanisms underlying these associations remain incompletely understood. Occupational shift schedules can disrupt temporal alignment between the central circadian pacemaker in the suprachiasmatic nucleus (SCN) and peripheral clocks, including those in the gastrointestinal tract. Intestinal circadian regulation involves epithelial, neuroendocrine, immune, and microbial processes that contribute to epithelial renewal, barrier function, gastrointestinal motility, nutrient handling, mucosal immunity, and host–microbiota interactions [4,7,8,9]. Circadian and neuroendocrine perturbations may therefore provide a biological context through which shift-work exposure influences gastrointestinal homeostasis, although the strength of evidence supporting individual downstream pathways varies considerably.

Circadian regulation of immune function may be an important component of this relationship. Major innate and adaptive immune-cell populations vary circadianly in trafficking and function, and experimental disruption of circadian clocks can alter inflammatory signaling and mucosal immune homeostasis [10,11,12,13,14,15,16,17,18,19,20]. Human studies of night-shift workers have also reported alterations in selected immune and inflammatory parameters [10,11,12,13,14,15]. However, direct longitudinal evidence demonstrating that immune dysregulation mediates the relationship between occupational shift work and gastrointestinal disease in humans remains limited. Similarly, circadian rhythms interact bidirectionally with the gut microbiota, whose composition and metabolic activity vary with feeding–fasting cycles and host circadian organization [15,21,22]. Evidence directly linking occupational shift work, microbiome alterations, and subsequent gastrointestinal disease is substantially more limited than the experimental evidence supporting these biological interactions.

Previous reviews have generally addressed shift-work epidemiology, circadian biology, gastrointestinal disorders, immune regulation, or the gut microbiota as partly separate domains. A synthesis that explicitly distinguishes direct occupational evidence from human observational associations, experimental mechanistic evidence, and biologically plausible but unproven relationships may help clarify what is established and where important evidence gaps remain.

Accordingly, this review critically examines a proposed integrative framework linking occupational shift exposure with circadian misalignment, neuroendocrine alterations, immune dysregulation, intestinal barrier dysfunction, microbial and metabolomic alterations, and gastrointestinal outcomes. The framework also considers bidirectional barrier–microbiota interactions and relevant behavioral mediators, including sleep and meal timing. It is intended as an evidence-organizing and hypothesis-generating model rather than as a fully demonstrated linear causal pathway in human shift-working populations. Particular attention is given to the source and strength of evidence supporting individual transitions, disease-specific epidemiological findings, and the extent to which proposed preventive strategies are supported by occupational/circadian evidence, general gastrointestinal evidence, or remain insufficiently tested in shift-working populations.

**Figure 1:**
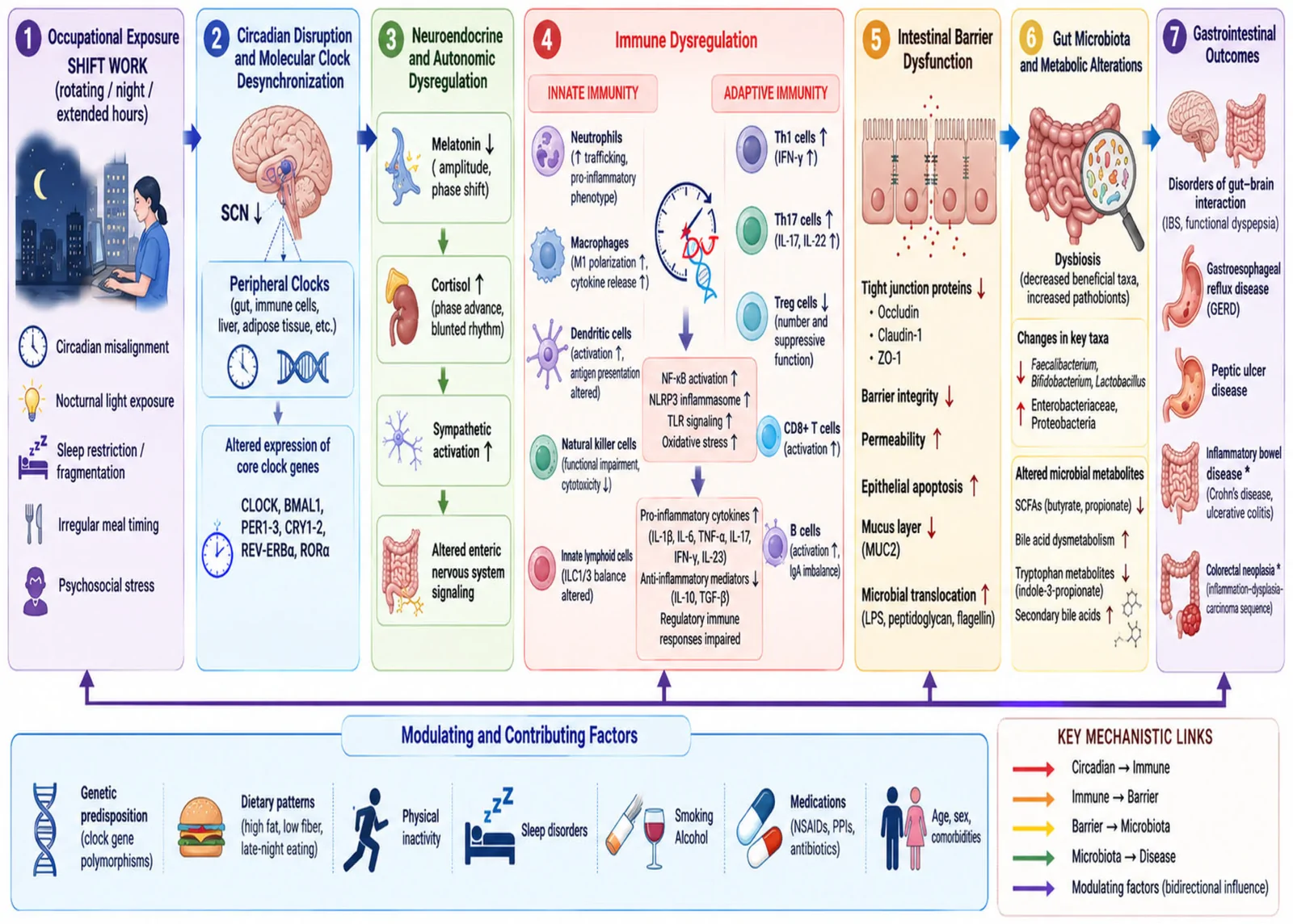
Integrated framework linking occupational shift work with circadian disruption and gastrointestinal health outcomes. Graphical illustration generated with the assistance of ChatGPT (OpenAI, GPT-5.6 Sol) based on detailed author-designed scientific concepts and subsequently reviewed and approved by the authors. Shift work acts as a complex occupational exposure characterized by circadian misalignment, nocturnal light exposure, sleep restriction, irregular meal timing, and psychosocial stress. These factors disrupt central and peripheral circadian organization and may interact with neuroendocrine alterations, immune dysregulation, intestinal barrier dysfunction, and gut microbiota and metabolic alterations, potentially contributing to gastrointestinal outcomes. The lower panel summarizes major host and environmental factors that may modulate these pathways, whereas the right panel highlights their translational relevance for occupational medicine. Solid arrows depict proposed or evidence-supported relationships within the framework rather than uniformly demonstrated sequential causal transitions, while the colored arrows in the key denote the principal biological interactions among circadian disruption, immune dysregulation, intestinal barrier dysfunction, gut microbiota alterations, and gastrointestinal outcomes. The diagram represents an integrative, hypothesis-generating framework and should not be interpreted as indicating equivalent evidentiary strength or established causality across all depicted relationships. Evidence ranges from direct observations in human shift workers and human observational studies to controlled experimental/animal studies and biologically plausible but currently hypothetical relationships, as summarized in Table 1. Bidirectional arrows indicate reciprocal interactions supported or proposed on the basis of the available evidence. Upward (↑) and downward (↓) arrows indicate increased and decreased biological activity or expression, respectively.

Figure 1 summarizes the conceptual framework. The arrows depict proposed biological relationships and do not imply equivalent evidentiary strength or established causality across all transitions.

## 2. Literature Search Strategy and Review Methodology

### 2.1. Literature Search and Evidence Synthesis

This narrative review synthesizes current evidence on the biological mechanisms linking occupational shift work and circadian disruption to gastrointestinal dysfunction and disease, with particular emphasis on neuroendocrine regulation, immune homeostasis, intestinal barrier integrity, gut microbiota, and their translational implications for occupational medicine. We conducted a structured literature search in PubMed/MEDLINE, Scopus, and Web of Science, focusing primarily on studies published between January 2020 and July 2026.

The search strategy combined Medical Subject Headings (MeSH), where applicable, and free-text keywords related to shift work, night work, rotating shift work, occupational exposure, circadian disruption, circadian misalignment, chronodisruption, sleep disruption, gastrointestinal disorders, irritable bowel syndrome, functional dyspepsia, gastroesophageal reflux disease, peptic ulcer disease, IBD, colorectal cancer, Helicobacter pylori, gut microbiota, intestinal permeability, epithelial barrier, immune dysregulation, inflammation, cytokines, oxidative stress, melatonin, cortisol, autonomic nervous system (ANS), microbial metabolites, chrononutrition, and occupational health. We used relevant combinations of these terms to identify studies addressing both mechanistic pathways and clinical gastrointestinal outcomes. We also manually screened the reference lists of relevant original studies, systematic reviews, and meta-analyses to identify additional eligible publications.

We prioritized recent original research articles, prospective and observational studies, systematic reviews, meta-analyses, and high-quality mechanistic and translational studies published in peer-reviewed journals. When available, we prioritized studies involving occupational cohorts and workers exposed to rotating or permanent night shifts. We included experimental and preclinical studies when they provided essential mechanistic evidence on circadian regulation of neuroendocrine and immune pathways, intestinal epithelial barrier function, or host–microbiota interactions. We retained landmark studies published before 2020 when they provided fundamental evidence that remains relevant to the current understanding of shift work, circadian biology, occupational health, or gastrointestinal pathophysiology.

We synthesized evidence narratively using a seven-stage mechanistic framework, progressing from occupational shift exposure through central and peripheral circadian clock disruption, neuroendocrine misalignment, circadian immune dysregulation, intestinal barrier dysfunction, gut microbial dysbiosis, and metabolic remodeling, ultimately culminating in clinically manifest gastrointestinal disease. We focused on circadian immune dysregulation as a central biological interface linking systemic chronodisruption with epithelial barrier impairment and microbial alterations. We critically interpreted clinical evidence alongside experimental findings to distinguish established epidemiological associations from biologically plausible mechanisms that remain insufficiently validated in occupational populations.

The review also examined emerging approaches relevant to occupational medicine, including individual risk stratification, circadian and inflammatory biomarkers, intestinal barrier markers, microbiome-derived metabolites, chrononutrition, mechanism-based preventive interventions, and multi-omics technologies. These findings were integrated into a conceptual framework for Precision Occupational Medicine, emphasizing the potential transition from conventional exposure- and symptom-based surveillance toward biomarker-informed risk assessment, early identification of biologically susceptible workers, and individualized gastrointestinal disease prevention.

This review was conducted as a narrative review and therefore did not follow the Preferred Reporting Items for Systematic Reviews and Meta-Analyses (PRISMA) statement. We did not perform a formal meta-analysis, quantitative evidence grading, or systematic risk-of-bias assessment of the included studies.

### 2.2. Use of Generative Artificial Intelligence

Generative artificial intelligence (GenAI), specifically ChatGPT (OpenAI, GPT-5.6 Sol), was used exclusively to assist in preparing the graphical illustrations in this review (Figure 1). The authors conceived the scientific concepts, biological mechanisms, figure organization, relationships among the individual components, and the visual content of each figure, and provided detailed, iterative instructions to generate graphical renderings that accurately reflected the evidence synthesized in the manuscript. The AI tool was used solely for visual illustration and graphical rendering and was not used for literature searching, study selection, evidence synthesis, data extraction, data analysis, interpretation of findings, or manuscript writing. The authors critically reviewed, scientifically verified, and approved all graphical outputs and took full responsibility for the scientific accuracy, integrity, and final content of the figures and the manuscript.

## 3. Mechanistic Framework Linking Shift Work to Gastrointestinal Diseases

### 3.1. Shift Work as a Multidimensional Occupational Exposure

Shift work has traditionally been classified by work schedule characteristics, including permanent night work, rotating shifts, evening shifts, split shifts, extended working hours, and other forms of non-standard employment. However, this administrative classification does not adequately capture the biological complexity of occupational exposure. Increasing evidence suggests that shift work should be regarded as a multidimensional occupational exposure, in which circadian misalignment may arise from the simultaneous interaction of nocturnal light exposure, recurrent sleep restriction and fragmentation, irregular meal timing, psychosocial stress, prolonged wakefulness, and disruption of normal work–rest cycles, rather than from altered working hours alone [1,23,24,25,26,27,28,29,30,31,32,33,34].

Although occupational and controlled human studies strongly support the association between night and rotating shift work and circadian misalignment, this relationship should not be interpreted as implying uniform or invariably demonstrated causality across all exposed workers. The magnitude of circadian disruption varies with schedule characteristics, the timing and intensity of nocturnal light exposure, sleep and meal timing, chronotype, and individual adaptive capacity. Accordingly, shift work is best regarded as an occupational exposure associated with a high likelihood of circadian disruption, while the degree of biological misalignment and its downstream consequences remain heterogeneous among individuals.

The multidimensional nature of this exposure is biologically relevant because its individual components may influence circadian organization through partly overlapping pathways. Artificial light at night can suppress nocturnal melatonin secretion and alter circadian entrainment, whereas irregular sleep–wake schedules may impair synchronization between the central circadian pacemaker in the SCN and peripheral molecular clocks. Irregular eating, fatigue, occupational stress, and recurrent misalignment between endogenous rhythms and externally imposed schedules may further modify neuroendocrine, metabolic, immune, and gastrointestinal responses [4,10,14,24,25,31,35]. Importantly, controlled experimental studies indicate that circadian misalignment and sleep restriction can exert partly independent physiological effects, supporting their consideration as related but distinct components of shift-work exposure [11,13,14,24,25,31].

Occupational characteristics further influence the magnitude of circadian disruption, including shift rotation direction, the frequency of consecutive night shifts, shift duration, recovery periods between shifts, workplace lighting, workload, and organizational factors. Evidence-based recommendations from the Working Time Society and occupational health experts emphasize that forward-rotating schedules, adequate recovery intervals, limiting consecutive night shifts, and optimized workplace lighting may substantially reduce circadian strain and improve worker health and safety [32,33,34]. Nevertheless, substantial interindividual variability remains, as chronotype, age, sex, genetic background, lifestyle behaviors, and pre-existing medical conditions further modify susceptibility to shift work [23,31,32,33,34].

This multidimensional exposure model provides the occupational context for the mechanistic framework examined in the following sections. Rather than assuming a uniform causal sequence, the framework considers how circadian and behavioral components of shift work may interact with neuroendocrine, immune, epithelial, microbial, and metabolic processes, while recognizing that evidence strength differs across these proposed relationships.

### 3.2. Occupational Work Environment as a Modifier of Biological Responses to Shift Work

Shift work occurs within a broader occupational environment that may amplify or buffer the biological effects of circadian disruption. Beyond work timing itself, psychosocial job characteristics—including workload and psychological demands, decision latitude or job control, effort–reward imbalance, social support, staffing adequacy, and opportunities for recovery—may influence physiological responses to non-standard work schedules. Prospective evidence indicates that adverse work-related psychosocial conditions are associated with small increases in inflammatory activity, although associations vary according to the specific exposure and biomarker and the available studies remain methodologically heterogeneous [36,37]. Consequently, workers exposed to similar shift schedules may experience different cumulative biological burdens depending on the organizational and psychosocial context in which those schedules occur.

Psychosocial occupational exposures may interact with circadian disruption through neuroendocrine and autonomic pathways. Sustained job demands combined with low control, insufficient recovery, or inadequate workplace support may contribute to repeated activation or dysregulation of stress-response systems, including the hypothalamic–pituitary–adrenal axis and sympathetic–adrenal–medullary/autonomic pathways [36,37,38]. Occupational studies have associated adverse working conditions with inflammatory biomarkers, while extended shift work and occupational stress have also been linked with alterations in autonomic regulation [36,37,38]. These pathways provide a biologically plausible connection with gastrointestinal motility, mucosal immune regulation, and epithelial homeostasis. Experimental and translational evidence indicates that psychosocial stress can influence intestinal barrier function through neuroendocrine, autonomic, immune, and mast-cell-related mechanisms; however, evidence that psychosocial stress consistently increases intestinal permeability in healthy humans remains limited and equivocal [39]. Conversely, greater job control, adequate recovery opportunities, and supportive work environments may buffer stress-related responses, although their capacity to prevent gastrointestinal barrier dysfunction specifically in shift workers remains unclear [37,40].

The physical demands of work may also modify the relationship. Shift work may occur in predominantly sedentary occupations characterized by prolonged sitting or, conversely, in physically demanding occupations involving prolonged standing, repetitive activity, heavy lifting, or sustained exertion. These exposure patterns should not be considered biologically equivalent. This distinction is consistent with the “physical activity paradox,” whereby leisure-time physical activity is generally associated with favorable health effects, whereas high occupational physical activity—particularly when prolonged, repetitive, or accompanied by insufficient recovery—may not confer equivalent benefits and may in some settings be associated with physiological strain and inflammatory responses [41,42,43]. Occupational evidence also suggests that both sedentary work and high-intensity occupational physical activity, particularly heavy physical tasks, may be associated with less favorable inflammatory profiles under certain exposure conditions [41].

Regular physical activity may also influence the gut microbiome and microbial metabolism, although available findings are heterogeneous. A recent systematic review and meta-analysis found no consistent effect of physical activity on microbial alpha-diversity, despite evidence of differences in microbial composition and functional pathways across activity patterns [44]. Accordingly, it would be premature to infer that occupational physical activity produces a specific beneficial or adverse gut microbial signature. The biological consequences of physical activity in shift workers are more likely to depend on its occupational versus leisure context, intensity, duration, associated postures and tasks, cardiorespiratory fitness, psychosocial demands, and opportunities for recovery [41,42,43,44].

These considerations extend the proposed framework beyond shift timing alone. Work schedule, psychosocial work organization, physical workload, recovery opportunities, and individual behavioral factors may interact to determine the effective occupational exposure each worker experiences. In the present framework, these factors are therefore regarded primarily as effect modifiers or potential buffering influences rather than additional obligatory stages of the mechanistic cascade. Their inclusion may help explain interindividual heterogeneity in circadian, neuroendocrine, immune, epithelial, microbial, and gastrointestinal responses among workers exposed to apparently similar shift schedules.

### 3.3. The Proposed Mechanistic Cascade

Researchers have traditionally studied the relationship between shift work and gastrointestinal disease through separate lines of research in occupational epidemiology, circadian biology, immunology, or gastroenterology. Although these studies have substantially advanced our understanding of individual pathogenic mechanisms, they have generally examined isolated components of a much broader biological process. Consequently, the available evidence remains fragmented, limiting how well we can integrate individual findings into a coherent pathway from occupational exposure to gastrointestinal outcomes.

To organize this fragmented evidence, we propose an integrative mechanistic framework linking occupational shift work with circadian, neuroendocrine, immune, epithelial, microbial, and gastrointestinal alterations (Figure 1). The framework connects evidence across biological levels rather than implying an established sequential causal pathway in shift-working populations.

Importantly, the proposed cascade should not be interpreted as a fully established causal sequence in human shift-working populations. The strength and directness of evidence vary substantially across individual transitions. To make this distinction explicit, evidence considered throughout this review was classified into four levels: (I) established in human shift workers, based on replicated occupational studies directly assessing the exposure and relevant biological or clinical endpoint; (II) supported by human observational evidence, including studies of shift workers or human circadian disruption in which associations are demonstrated but causal mediation remains uncertain; (III) supported mainly by controlled experimental, laboratory, or animal studies that provide mechanistic evidence but have limited direct occupational validation; and (IV) biologically plausible but currently hypothetical, where the proposed transition is inferred from converging mechanistic evidence without direct demonstration in shift-working populations. Accordingly, Figure 1 represents an integrative and hypothesis-generating framework rather than a clinically validated linear causal pathway. In particular, direct longitudinal studies simultaneously tracking circadian, neuroendocrine, immune, epithelial barrier, microbial, and gastrointestinal outcomes in occupational cohorts remain scarce.

To make these differences in evidentiary strength transparent, Table 1 maps each major transition within the proposed cascade to the predominant type of supporting evidence and the corresponding evidence category. This evidence hierarchy distinguishes relationships directly demonstrated in occupational populations from those supported primarily by observational, experimental, or hypothesis-generating mechanistic data.

Within this framework, occupational shift exposure is considered in relation to circadian misalignment and downstream neuroendocrine alterations, followed by potential changes in immune regulation, intestinal barrier function, host–microbiota interactions, and gastrointestinal outcomes. Immune dysregulation is examined as an important mechanistic interface within this framework; however, its mediating role across the complete pathway has not been demonstrated longitudinally in human shift workers. Similarly, barrier dysfunction and microbial alterations are treated as potentially reciprocal processes rather than obligatory sequential events.

Importantly, the proposed cascade should not be viewed as strictly unidirectional. Multiple bidirectional interactions exist between intestinal barrier integrity and gut microbial composition, and behavioral and psychosocial factors—including sleep quality, dietary habits, occupational stress, physical activity, smoking, alcohol consumption, medication use, and individual susceptibility factors such as chronotype, age, and genetic background—may modify the proposed relationships at virtually every stage of the pathway. Consequently, the cascade should be regarded as a dynamic biological network in which several mechanisms may operate concurrently and interact reciprocally rather than following an invariant sequence.

The following sections therefore evaluate the individual components of this framework with explicit attention to the source, directness, and limitations of the supporting evidence.

## 4. Biological Stages of the Shift Work–Gastrointestinal Disease Cascade

Building on the evidence hierarchy outlined in Section 3.2, the following sections examine the individual biological components of the proposed framework. Each stage is discussed with attention to the source and strength of supporting evidence, distinguishing findings from occupational and human observational studies from those derived predominantly from experimental or animal models. Particular emphasis is placed on areas in which direct evidence from shift-working populations remains limited.

### 4.1. Stage 1—Occupational Shift Exposure: The Initial Biological Trigger

Within the proposed framework, occupational shift work represents the initial exposure rather than a demonstrated biological trigger of gastrointestinal disease. Chronic rotating and permanent night-shift schedules may combine recurrent circadian misalignment with artificial light at night, irregular eating, prolonged wakefulness, sleep restriction, and psychosocial stress. The intensity and combination of these exposures vary considerably across work schedules and individuals [2,3,23,24,25,26,27,28,29,30,31,32,33,34].

From an occupational medicine perspective, the biological consequences of shift work depend not only on night work but also on the cumulative characteristics of the work schedule. Several occupational variables—including the frequency of night shifts, the number of consecutive night shifts, rotation direction, shift duration, recovery intervals, workplace lighting, workload, and years of occupational exposure—may influence the magnitude of circadian misalignment and associated physiological responses [24,25,31,32,33,34]. Forward-rotating schedules, limiting consecutive night shifts, providing adequate recovery periods, and optimizing workplace lighting have been proposed to facilitate circadian adaptation and reduce physiological strain, as reflected in occupational scheduling recommendations [32,33].

These schedule-related exposures act through several environmental and behavioral time cues. Nocturnal light exposure can suppress melatonin secretion and alter circadian phase, whereas irregular feeding schedules may desynchronize peripheral metabolic rhythms from the central pacemaker. Changes in sleep timing, sleep restriction, and occupational stress may additionally influence neuroendocrine and autonomic regulation [4,10,14,24,25,31,35]. However, the magnitude of these responses varies across individuals and exposure patterns, and their combined contribution to gastrointestinal outcomes has not been established as a uniform causal sequence in occupational populations.

Epidemiological studies indicate that shift work is associated with an increased burden of several gastrointestinal symptoms and disorders, although the strength of evidence differs by outcome [2,3,5,6,45]. The most consistent evidence concerns IBS and gastrointestinal symptom burden, whereas associations with other disease categories are less consistently demonstrated. Prospective cohort evidence has further identified exposure–response relationships between the duration and frequency of night-shift work and incident IBS [6]. These findings support an occupational association but do not, by themselves, establish that circadian disruption mediates the observed gastrointestinal risk.

Importantly, not all exposed workers develop clinically significant gastrointestinal disease, indicating that occupational exposure alone cannot explain disease susceptibility. Individual factors—including chronotype, age, sex, genetic background, lifestyle behaviors, psychological resilience, and pre-existing metabolic or inflammatory conditions—may modify responses to shift-work exposure and contribute to the interindividual variability observed across epidemiological studies [23,31,32,33].

Accordingly, Stage 1 defines the occupational exposure context from which the subsequent circadian alterations considered in this framework may emerge. The next section examines the evidence linking shift-work exposure with disruption of central and peripheral circadian organization.

### 4.2. Stage 2—Central and Peripheral Circadian Clock Disruption

A major biological correlate of chronic occupational shift work is disruption of circadian organization, although the magnitude of this disturbance varies considerably among exposed workers. Under physiological conditions, the SCN of the anterior hypothalamus orchestrates circadian rhythms as the master circadian pacemaker, synchronizing peripheral clocks across virtually all tissues and organs. This hierarchical organization ensures temporal coordination of endocrine secretion, immune surveillance, metabolic activity, cellular proliferation, and gastrointestinal function in predictable 24-h oscillations [4,8].

At the molecular level, circadian rhythmicity arises from interconnected transcriptional–translational feedback loops centered on the core clock genes CLOCK, BMAL1 (ARNTL), PERIOD (PER1–PER3), CRYPTOCHROME (CRY1–CRY2), REV-ERBα/β, and RORα/γ. The CLOCK–BMAL1 heterodimer drives transcription of the PER and CRY genes, and their protein products then inhibit their own transcription, producing self-sustained oscillations with approximately 24-h periodicity. Additional regulatory loops involving REV-ERB and ROR nuclear receptors further stabilize these oscillations and coordinate the rhythmic expression of hundreds of downstream clock-controlled genes involved in inflammation, metabolism, epithelial integrity, and immune regulation [4].

Although the SCN is primarily entrained by the environmental light–dark cycle, peripheral clocks respond to a broader range of physiological synchronizers, including feeding behavior, glucocorticoid secretion, ANS activity, body temperature, physical activity, and microbe-derived metabolites [4,8,9]. Shift-work schedules can create conflicting temporal cues: nocturnal light exposure may shift the central circadian phase, while changes in meal timing, sleep–wake behavior, and activity provide additional signals to peripheral oscillators. Controlled human and occupational studies support circadian misalignment under night and rotating work schedules, but the degree of central–peripheral desynchronization depends on exposure characteristics and individual adaptation.

The gastrointestinal tract is one of the most complex peripheral circadian systems. Functional molecular clocks have been identified in intestinal epithelial cells, Paneth cells, goblet cells, enteric neurons, stromal cells, and resident immune-cell populations. Through coordinated rhythmic gene expression, these peripheral clocks regulate epithelial renewal, intestinal permeability, mucus secretion, antimicrobial peptide production, nutrient absorption, gastrointestinal motility, bile acid metabolism, and mucosal immune surveillance [7,8,9,16,20]. Maintaining temporal synchrony among these biological processes is essential for preserving intestinal homeostasis.

Experimental studies provide strong mechanistic evidence that disruption of local circadian clocks can impair intestinal homeostasis. In murine models, intestinal epithelial-specific deletion of Bmal1 abolishes rhythmic expression of genes involved in antimicrobial defense, epithelial regeneration, and inflammatory regulation, thereby increasing susceptibility to intestinal inflammation [9]. Additional studies have shown that circadian rhythm disturbance alters epithelial transcriptional programs, promotes inflammatory signaling, and modifies host responses to the intestinal microbiota, confirming that disruption of peripheral clock function alone is sufficient to compromise gastrointestinal homeostasis [8,15,20]. These findings demonstrate experimental causality at the level of intestinal clock disruption but should not be interpreted as direct evidence that equivalent molecular alterations occur to the same extent in human shift workers.

Thus, circadian disruption provides a biologically plausible interface between occupational shift exposure and downstream systemic alterations, but evidence differs across levels of analysis. Human shift-work and controlled circadian studies support alterations in circadian timing, whereas much of the evidence linking local molecular-clock disruption to intestinal dysfunction derives from experimental models. This distinction is important when extrapolating from molecular mechanisms to occupational gastrointestinal disease.

The next stage of the proposed framework considers neuroendocrine signaling, particularly melatonin, cortisol, and autonomic regulation, as systemic pathways through which circadian misalignment may influence downstream physiological processes.

### 4.3. Stage 3—Neuroendocrine Misalignment: Translating Circadian Disruption into Systemic Biological Signals

Circadian misalignment may be accompanied by alterations in neuroendocrine rhythmicity that provide systemic signals linking central circadian organization with peripheral physiology. Under physiological conditions, the SCN coordinates peripheral organs through rhythmic endocrine secretion and autonomic signaling. These outputs influence immune surveillance, epithelial function, gastrointestinal motility, nutrient metabolism, and other processes relevant to gastrointestinal homeostasis [4,10,24,25,31,35]. In shift-working populations, alterations in melatonin and cortisol rhythms have been documented, although their magnitude varies according to work schedule, light exposure, sleep timing, and individual adaptation.

#### 4.3.1. Melatonin Signaling: The Principal Circadian Endocrine Messenger

Among the endocrine mediators affected by shift work, melatonin represents the best-characterized biological signal linking environmental light exposure to circadian physiology. Synthesized by the pineal gland predominantly during the biological night, melatonin synchronizes peripheral circadian clocks while simultaneously exerting antioxidant, anti-inflammatory, and immunomodulatory effects. Beyond regulating sleep–wake cycles, melatonin influences mitochondrial function, leukocyte activity, cytokine production, epithelial barrier integrity, intestinal motility, and microbial rhythmicity, thereby providing several biologically plausible pathways relevant to gastrointestinal homeostasis [4,10,35].

Exposure to artificial light during nighttime can suppress melatonin synthesis and alter the timing and amplitude of its circadian rhythm. Occupational studies, including the HORMONIT study, have documented alterations in melatonin secretion among rotating night-shift workers, supporting a direct relationship between night-work exposure and altered circadian endocrine signaling [10,35]. Experimental and non-occupational evidence further indicates that reduced melatonin signaling may influence antioxidant defense, oxidative stress, epithelial protection, and inflammatory regulation [10,14,35]. However, direct evidence demonstrating that altered melatonin secretion mediates gastrointestinal disease in shift workers remains limited.

#### 4.3.2. Dysregulation of the Hypothalamic–Pituitary–Adrenal Axis

Alongside melatonin suppression, chronic shift work profoundly affects the hypothalamic–pituitary–adrenal axis, another major circadian-controlled effector system. Physiologically, cortisol secretion follows a robust circadian rhythm, characterized by an early-morning peak followed by a gradual decline throughout the day. This rhythmic glucocorticoid signaling helps regulate immune-cell trafficking, inflammatory responses, energy metabolism, and adaptation to environmental stressors [4,24,25,31].

Shift work and experimentally induced circadian misalignment have been linked to changes in the timing and amplitude of cortisol secretion [4,24,25,31]. Because glucocorticoids regulate cytokine production, leukocyte migration, epithelial permeability, and inflammatory resolution, altered cortisol rhythmicity provides a plausible pathway through which circadian misalignment could influence mucosal immune homeostasis. Nevertheless, evidence directly linking shift-work-related cortisol alterations to subsequent gastrointestinal disease remains insufficient.

#### 4.3.3. Autonomic Nervous System Dysregulation

The ANS constitutes another communication pathway through which circadian organization can influence peripheral organ function. Sympathetic and parasympathetic activity normally exhibits marked circadian oscillations that coordinate gastrointestinal motility, intestinal secretion, mucosal blood flow, and immune function. Circadian misalignment and sleep disruption may alter sympathovagal balance, although direct characterization of these changes in relation to gastrointestinal outcomes among shift workers remains limited [4,31].

Mechanistically, sympathetic adrenergic signaling can modulate inflammatory responses, whereas vagal signaling participates in the cholinergic anti-inflammatory reflex. These pathways provide a biological rationale for interactions among circadian disruption, autonomic regulation, intestinal function, and mucosal immunity; however, much of the evidence supporting these downstream relationships derives from experimental or non-occupational studies rather than longitudinal studies of shift-working populations [4,31].

#### 4.3.4. Neuroendocrine–Immune Crosstalk: The Biological Bridge to Immune Dysregulation

Melatonin, glucocorticoids, autonomic signaling, and molecular circadian pathways interact with multiple components of innate and adaptive immunity, including leukocyte trafficking and cytokine regulation [4,10,11,12,13,14,35]. Experimental evidence therefore supports neuroendocrine–immune crosstalk as a plausible interface between circadian misalignment and altered immune function. Human shift-work studies provide evidence of neuroendocrine and immune alterations, but they have not demonstrated the full mediating sequence from occupational exposure through neuroendocrine disruption to persistent immune dysregulation longitudinally.

Accordingly, neuroendocrine misalignment is considered an intermediate component of the proposed framework rather than an obligatory causal step. The following section examines circadian immune dysregulation in greater detail, while distinguishing direct evidence from shift-working populations from mechanistic evidence derived from experimental models.

### 4.4. Stage 4—Circadian Immune Dysregulation: The Central Biological Hub Connecting Shift Work with Gastrointestinal Disease

Immune function is closely integrated with circadian and neuroendocrine regulation. Under physiological conditions, leukocyte trafficking, cytokine production, antigen presentation, and several components of innate and adaptive immunity vary over time [4,10,11,12,13,14,35]. Experimental circadian disruption can alter these processes, and human shift-work studies have identified changes in selected inflammatory and immune parameters. However, direct evidence demonstrating that immune dysregulation mediates the relationship between occupational shift work and gastrointestinal disease remains limited.

#### 4.4.1. Circadian Regulation of Innate Immunity

Innate immune cells exhibit substantial circadian variation in trafficking and function. Molecular clocks and neuroendocrine mediators, including glucocorticoids, melatonin, and autonomic signals, help regulate the timing of neutrophils, monocytes, macrophages, dendritic cells, natural killer (NK) cells, and innate lymphoid cells (ILCs) [4,11,12,13]. These rhythms influence leukocyte migration, pathogen recognition, cytokine production, and tissue repair.

Among circulating leukocytes, neutrophils display marked circadian variation in mobilization, endothelial adhesion, and migratory capacity. Monocytes and tissue macrophages similarly exhibit rhythmic inflammatory responsiveness, whereas dendritic cells, NK cells, and ILCs show time-dependent changes in functions relevant to antigen presentation, cytotoxicity, and tissue immune surveillance [4,11,13].

Human studies of night-shift workers have reported alterations in circulating inflammatory markers and selected immune parameters [10,11,13]. These findings support an association between occupational circadian disruption and altered immune regulation, although sleep restriction, psychosocial stress, workload, and other co-exposures complicate attribution specifically to circadian misalignment. Experimental studies provide stronger mechanistic evidence that disruption of circadian organization can modify inflammatory responsiveness, cytokine signaling, oxidative balance, and inflammatory resolution [4,11,14].

Within the gastrointestinal tract, macrophages, dendritic cells, and ILCs participate in epithelial maintenance, microbial sensing, and mucosal immune regulation. Experimental studies indicate that disruption of circadian signaling in these cellular compartments can alter intestinal immune homeostasis [8,9,15,16,17,18,19,20]. However, equivalent changes have not been comprehensively demonstrated in the intestinal tissues of human shift workers.

#### 4.4.2. Circadian Regulation of Adaptive Immunity

Circadian regulation extends to adaptive immune function, with molecular clocks, glucocorticoid signaling, sympathetic innervation, and cytokine networks influencing T- and B-lymphocyte trafficking and activity [4,12,13].

Researchers have focused on the balance between pro-inflammatory and regulatory T-cell subsets. Experimental studies have demonstrated that circadian disruption alters CD4^+^ T-cell activation, modulates interferon-γ production, and can influence regulatory pathways relevant to intestinal immune tolerance [19]. Clock-controlled transcriptional pathways have also been implicated in the differentiation and function of regulatory and effector T-cell populations [4,16,17,18,19,20]. These findings provide mechanistic evidence for circadian regulation of adaptive immunity, but the contribution of individual lymphocyte subsets in human shift workers remains incompletely characterized.

Human occupational studies provide more limited evidence. Epigenetic alterations involving immune-related genes and changes in selected circulating immune parameters have been reported among night-shift workers [11,12,13]. These observations are consistent with altered immune regulation but do not establish persistent adaptive immune reprogramming or its causal contribution to gastrointestinal disease.

In the gastrointestinal tract, adaptive immune regulation is essential for maintaining tolerance to luminal antigens and commensal microorganisms. Experimental evidence therefore provides a plausible connection between altered circadian immune regulation and mucosal inflammation, although this pathway requires direct validation in occupational populations [16,17,18,19,20].

#### 4.4.3. Molecular Mechanisms of Circadian Inflammatory Activation

Circadian clocks interact with several intracellular pathways involved in inflammatory regulation, including NF-κB signaling, inflammasome activity, cytokine production, and redox homeostasis [4,15,16,17,18,19,20]. Much of the evidence defining these molecular interactions derives from experimental systems and disease models rather than occupational cohorts.

Among the best-characterized mechanisms is the interaction between circadian clock components and NF-κB signaling. BMAL1, REV-ERBα, and related clock-controlled pathways can modulate inflammatory transcription, providing a mechanistic basis for time-dependent variation in mediators such as IL-1β, IL-6, TNF-α, IFN-γ, and IL-17 [4,16,17,18,19,20]. Experimental disruption of clock function can enhance pro-inflammatory signaling, but direct demonstration of sustained NF-κB activation as a consequence of occupational shift work remains limited.

A second key mechanism involves the NLRP3 inflammasome, which regulates IL-1β and IL-18 maturation. Experimental evidence links circadian and metabolic disturbances with inflammasome activity through mechanisms involving oxidative stress, mitochondrial function, and metabolic signaling [15,16,17,18,19,20]. These findings support mechanistic plausibility, particularly in experimental models of intestinal inflammation, but direct evidence in shift-working populations is insufficient.

Oxidative stress may represent an additional interacting mechanism. Night-shift populations have shown altered oxidative stress and antioxidant biomarkers, and experimental evidence indicates reciprocal interactions among redox imbalance, NF-κB signaling, inflammasome activity, and inflammatory pathways [8,15]. Nevertheless, these findings do not establish a specific causal sequence from shift work to oxidative stress, immune dysregulation, and gastrointestinal injury.

Overall, these molecular pathways provide experimental support for interactions between circadian regulation and inflammatory signaling. However, their contribution to gastrointestinal disease among human shift workers remains to be established prospectively.

#### 4.4.4. Human Evidence of Circadian Immune Dysregulation in Shift Workers

Human occupational studies provide evidence that night and rotating shift work are associated with alterations in selected inflammatory, immune, oxidative, and epigenetic parameters [10,11,12,13]. However, these studies are heterogeneous in design, exposure assessment, sampling time, biomarker selection, and control of sleep- and lifestyle-related confounders, limiting causal interpretation.

The HORMONIT study reported alterations in cellular immune parameters, along with changes in melatonin and endocrine profiles, among rotating night-shift workers [10,35]. These findings demonstrate concurrent neuroendocrine and immune alterations but do not establish the direction or mediation of these relationships.

Studies of healthcare professionals have likewise identified associations between night-shift exposure and inflammatory profiles related to sleep debt and social jet lag [11]. Epigenetic analyses have reported differential DNA methylation involving immune-related genes among night-shift workers [12], while other occupational studies have identified changes in selected immune parameters [13].

Taken together, these studies support an association between shift work and altered immune regulation in humans. They do not, however, demonstrate that these immune changes become chronically self-sustaining or mediate subsequent intestinal barrier dysfunction and gastrointestinal disease. Longitudinal occupational studies integrating repeated circadian, immune, and gastrointestinal measurements are needed to test this proposed pathway directly.

#### 4.4.5. From Circadian Immune Dysregulation to Intestinal Barrier Function

Experimental and translational studies indicate extensive interactions between inflammatory signaling and intestinal epithelial integrity. Pro-inflammatory cytokines, oxidative stress, and altered immune-cell activity can influence tight-junction organization, epithelial renewal, mucus production, and permeability, while loss of epithelial compartmentalization can increase mucosal immune cells’ exposure to microbial products [15,16,17,18,19,20]. These reciprocal interactions provide a mechanistic basis for linking altered immune regulation with intestinal barrier dysfunction.

Importantly, these processes may form a self-reinforcing immune–barrier–microbiota loop: inflammatory signaling can impair epithelial integrity, increased permeability may facilitate mucosal exposure to or translocation of microbial products, and these microbial signals may in turn amplify innate and adaptive inflammatory responses. Counter-regulatory mechanisms—including epithelial repair, mucus and antimicrobial defenses, regulatory immune responses, and beneficial microbial metabolites—may buffer this cycle and help restore intestinal homeostasis. Thus, progression toward persistent dysfunction is not inevitable but may depend on the balance between reinforcing and compensatory mechanisms.

However, most evidence supporting this transition derives from experimental models or studies of established gastrointestinal inflammation rather than prospective studies of shift workers. It therefore remains uncertain whether the immune alterations observed in occupational populations are sufficient to produce clinically relevant barrier dysfunction or whether additional behavioral, dietary, microbial, and host factors are required.

Barrier dysfunction should consequently be regarded as a potential interface between circadian–immune disruption and altered host–microbiota interactions rather than as an inevitable downstream consequence of shift work. Increased permeability and impaired epithelial compartmentalization may facilitate microbial translocation and reinforce mucosal inflammation, providing the transition to the intestinal barrier processes examined in the following section.

To help interpret the complex interactions described above, Table 2 summarizes the principal immune-cell populations involved in circadian immune regulation, their physiological functions, the alterations reported or proposed under circadian disruption, the predominant source of supporting evidence, and their potential implications for gastrointestinal homeostasis.

### 4.5. Stage 5—Intestinal Barrier Dysfunction: The Gateway to Gastrointestinal Disease

The intestinal barrier represents one of the most complex biological interfaces in the human body, separating the host from trillions of microorganisms while simultaneously allowing efficient nutrient absorption and maintaining immune tolerance. Rather than functioning as a passive physical boundary, the intestinal barrier is increasingly recognized as an integrated structural, immunological, and metabolic system comprising epithelial cells, mucus layers, antimicrobial peptides, resident immune cells, tight junction complexes, vascular and neural networks, and the intestinal microbiota. Preservation of this highly coordinated barrier is essential for maintaining gastrointestinal homeostasis and preventing uncontrolled immune activation [8,9,15,16,17,18,19,20].

Structurally, the intestinal barrier consists of several complementary defense layers. The outer mucus layer, produced primarily by goblet cells, limits direct microbial contact with epithelial cells while serving as a reservoir for antimicrobial molecules. Beneath the mucus layer, a continuous monolayer of intestinal epithelial cells forms the principal physical barrier, with selective paracellular permeability regulated by tight junction proteins, including claudins, occludin, junctional adhesion molecules, and zonula occludens (ZO)-1 and ZO-2. Paneth cells contribute to mucosal defense by secreting antimicrobial peptides such as α-defensins and lysozyme, while resident immune cells in the lamina propria continuously monitor luminal antigens and maintain tolerance toward commensal microorganisms [8,9,16,17,18,19,20].

Circadian rhythms tightly regulate epithelial integrity. Functional molecular clocks expressed in intestinal epithelial cells coordinate epithelial proliferation, stem-cell renewal, mucus secretion, tight-junction assembly, nutrient transport, and epithelial repair in response to predictable daily oscillations. Experimental disruption of intestinal clock genes, particularly BMAL1, has been shown to impair epithelial regeneration, alter antimicrobial peptide expression, and increase susceptibility to intestinal inflammation, providing evidence that local circadian regulation contributes to barrier maintenance [8,9,16,20]. These findings demonstrate mechanistic effects in experimental systems but should not be interpreted as direct evidence of equivalent barrier alterations in occupational shift workers.

Autophagy and endoplasmic reticulum (ER) stress represent additional cellular pathways through which circadian disruption may influence intestinal epithelial homeostasis. Experimental evidence indicates that core circadian-clock components can interact with autophagic regulation in intestinal epithelial cells. In a recent study, circadian rhythm disruption and reduced BMAL1 expression were associated with impaired intestinal barrier function and increased susceptibility to colitis, whereas BMAL1-mediated activation of autophagy contributed to epithelial-cell survival and preserved tight-junction integrity [46]. These findings suggest that autophagy may function as a cellular homeostatic mechanism linking local circadian regulation with epithelial maintenance. However, this evidence derives predominantly from experimental models, and direct demonstration of a circadian–autophagy–barrier pathway in human shift workers is currently lacking.

Autophagy also interacts closely with ER stress and the unfolded protein response (UPR), particularly in highly secretory intestinal epithelial populations such as Paneth and goblet cells. These cells require efficient protein folding, secretion, and quality-control mechanisms and are therefore particularly sensitive to disturbances in ER homeostasis. Autophagy can compensate for ER stress by removing damaged organelles and misfolded proteins, whereas defective autophagy may promote persistent ER stress, inflammatory signaling, and epithelial dysfunction [47,48]. Recent experimental evidence in colonic goblet cells demonstrated that autophagy-mediated alleviation of ER stress promotes mucus secretion and formation of a less penetrable mucus barrier, with downstream effects on microbial composition and susceptibility to intestinal inflammation [48]. Conversely, sleep deprivation has been shown experimentally to activate ER stress in goblet cells, reduce goblet-cell numbers and mucin production, decrease tight-junction protein expression, and impair the intestinal mucosal barrier [49]. Paneth cells are similarly dependent on coordinated autophagy and UPR signaling for antimicrobial peptide secretion, organelle homeostasis, and survival, and disruption of these pathways can facilitate inflammatory responses and microbial dysregulation [47,50]. Thus, autophagy–ER stress crosstalk provides a plausible additional interface among circadian disruption, epithelial secretory-cell dysfunction, barrier impairment, and host–microbiota interactions. Nevertheless, its specific contribution to gastrointestinal dysfunction associated with occupational shift work remains hypothetical and requires direct human validation.

Experimental and translational studies also indicate that inflammatory signaling, oxidative stress, and disrupted local circadian regulation can affect epithelial renewal, mucus production, tight-junction organization, and intestinal permeability [15,16,17,18,19,20]. Pro-inflammatory cytokines, including TNF-α, IL-1β, IL-6, IFN-γ, and IL-17, can modify epithelial and tight-junction function, while oxidative and mitochondrial disturbances may further impair epithelial integrity. However, direct longitudinal evidence linking the immune alterations observed in shift workers to subsequent clinically relevant intestinal barrier dysfunction remains limited.

Increased intestinal permeability can, in turn, increase mucosal exposure to microbial products, including lipopolysaccharide, peptidoglycans, and other microbe-associated molecular patterns. Recognition of these signals by pattern-recognition receptors can reinforce inflammatory pathways [15,16,17,18,19,20]. Conversely, inflammation and microbial alterations can further compromise epithelial integrity, supporting a reciprocal relationship among barrier function, mucosal immunity, and the intestinal microbiota rather than a strictly unidirectional sequence.

Accordingly, intestinal barrier dysfunction should be considered a biologically plausible interface within the proposed framework rather than an obligatory intermediate step between occupational shift work and gastrointestinal disease. Although experimental evidence linking circadian and inflammatory disturbances with epithelial dysfunction is substantial, direct demonstration of this pathway in shift-working populations remains insufficient.

Table 3 summarizes the principal structural components of the intestinal barrier, their physiological functions, the alterations reported under circadian or inflammatory disruption, the predominant source of supporting evidence, and their potential gastrointestinal implications.

### 4.6. Stage 6—Gut Microbial Dysbiosis and Metabolic Remodeling: Amplification of the Inflammatory Cascade

Gut microbial composition and metabolic activity are closely interconnected with circadian organization, dietary timing, intestinal barrier function, and mucosal immunity. Accordingly, the microbiota should not be regarded simply as the terminal downstream component of a linear cascade. Rather, it participates in a reciprocal immune–barrier–microbiota network in which inflammation may impair epithelial integrity, barrier dysfunction may increase host exposure to microbial products, and altered microbial composition or metabolism may further influence epithelial and immune responses [7,8,15,16,17,18,19,20,21,22]. At the same time, epithelial repair mechanisms, mucus and antimicrobial defenses, regulatory immune pathways, homeostatic microbial metabolites, and the ecological resilience of microbial communities may counterbalance these reinforcing interactions and facilitate recovery toward intestinal homeostasis. Within the proposed framework, microbial alterations are therefore considered a dynamically interacting component rather than an obligatory downstream consequence of barrier dysfunction. Under physiological conditions, the intestinal microbiota exists in a dynamic symbiotic relationship with the host, contributing to nutrient metabolism, vitamin synthesis, immune maturation, epithelial renewal, colonization resistance, and maintenance of mucosal homeostasis. Microbial composition and metabolic activity also vary temporally with host circadian rhythms and feeding–fasting cycles [7,8,21,22].

Feeding behavior, intestinal motility, bile acid secretion, hormonal rhythms, and epithelial nutrient availability contribute to temporal variation within the intestinal microbial ecosystem. Experimental disruption of circadian organization or feeding schedules can modify these microbial rhythms, providing mechanistic support for interactions between host circadian biology and the gut microbiome [21,22].

Experimental studies have reported changes in microbial rhythmicity, diversity, taxonomic composition, and metabolic function following circadian disruption or altered feeding schedules [21,22]. However, individual microbial signatures vary substantially according to experimental model, diet, host characteristics, geography, and analytical methodology. Human studies of shift workers provide emerging evidence of microbiome differences associated with non-standard work schedules, but available cohorts remain relatively small and heterogeneous, and researchers have not established a reproducible shift-work-specific microbial signature. Direct causal inference from occupational shift work to persistent intestinal dysbiosis therefore remains premature.

Importantly, the biological consequences of dysbiosis extend far beyond alterations in microbial composition. Attention has increasingly shifted toward functional microbial metabolism, as microbial-derived metabolites are critical signaling molecules that regulate epithelial integrity, immune responses, and systemic metabolism. Among these metabolites, short-chain fatty acids (SCFAs)—particularly acetate, propionate, and butyrate—play central roles in maintaining epithelial barrier function by serving as energy substrates for colonocytes, promoting mucus production, strengthening tight-junction integrity, and supporting regulatory immune pathways. Experimental circadian and dietary perturbations have been associated with alterations in SCFA-producing microbial communities and metabolite profiles, although equivalent persistent changes have not been consistently demonstrated in occupational shift workers [21,22].

Beyond SCFAs, gut microorganisms regulate several additional metabolite classes with important immunological functions. Secondary bile acids signal through the farnesoid X receptor and the Takeda G protein receptor 5, influencing epithelial regeneration, inflammatory responses, and microbial ecology. Likewise, microbial metabolism of dietary tryptophan generates indole derivatives that activate the aryl hydrocarbon receptor, thereby contributing to epithelial barrier maintenance, mucosal immune tolerance, and IL-22 production. Alterations in these metabolic pathways may plausibly link circadian and dietary disruption to intestinal homeostasis, but direct occupational evidence remains limited [21,22].

Experimental evidence further supports reciprocal interactions among microbial composition, metabolite production, epithelial integrity, and mucosal immune signaling [15,16,17,18,19,20,21,22]. However, whether these interactions develop into persistent self-reinforcing loops in human shift workers, and the extent to which compensatory mechanisms preserve homeostasis, remain unknown.

Table 4 summarizes the principal microbial metabolites relevant to intestinal homeostasis, their physiological functions, the alterations reported under circadian or microbial disruption, the predominant evidence base, and their potential gastrointestinal implications.

Overall, microbiome and metabolomic findings provide substantial mechanistic plausibility but comparatively limited direct occupational evidence. Longitudinal studies combining repeated assessment of work schedules, circadian phase, diet and meal timing, microbiome composition, metabolomics, and gastrointestinal outcomes are required to determine whether microbial changes mediate the association between shift work and gastrointestinal disease.

### 4.7. Stage 7—Clinical Gastrointestinal Consequences: From Circadian Disruption to Disease

The clinical relevance of the proposed framework depends ultimately on whether occupational shift work is associated with measurable gastrointestinal outcomes in human populations. Importantly, mechanistic plausibility should not be equated with demonstrated disease causality. Epidemiological evidence varies substantially across gastrointestinal conditions, and the extent to which circadian, neuroendocrine, immune, epithelial, or microbial alterations mediate these associations remains largely unresolved.

Importantly, the epidemiological evidence supporting an association with occupational shift work is not equivalent across gastrointestinal disease categories. The most consistent evidence concerns disorders of gut–brain interaction, particularly IBS, where meta-analyses and large prospective cohorts have shown associations with night or rotating shift work, including exposure–response relationships with shift duration and frequency [5,6,51]. Evidence for functional dyspepsia is less consistent, whereas direct occupational epidemiological evidence for IBD remains limited [51]. Similarly, although shift work has been investigated in relation to colorectal neoplasia, findings across cohort studies and meta-analyses are heterogeneous and do not establish a consistent association [51,52]. Accordingly, the discussion below distinguishes epidemiologically supported associations from disease links that currently rely predominantly on mechanistic plausibility or experimental evidence.

Among the gastrointestinal outcomes examined in shift-working populations, the most consistent epidemiological evidence concerns disorders of gut–brain interaction (DGBIs), particularly IBS [3,5,6,45]. Meta-analytic evidence indicates a significantly higher prevalence of IBS among shift workers [3,5,45], while recent large prospective cohort data have further demonstrated an increased risk of incident IBS associated with night-shift work, with higher risks observed with increasing duration and frequency of night-shift exposure [6]. By contrast, evidence for functional dyspepsia is less consistent. Although earlier meta-analytic evidence did not demonstrate a statistically significant association between shift work and functional dyspepsia [5], more recent cross-sectional evidence suggests a substantial burden of Rome IV-defined functional dyspepsia among night-shift workers [53]. These findings support further investigation but do not yet establish a prospective exposure–disease relationship.

Gastroesophageal reflux symptoms and PUD have also been reported more frequently in some shift-working populations [2,3]. Potential contributing factors include altered meal timing, nocturnal eating, sleep disturbance, autonomic regulation, and other behavioral or physiological co-exposures. However, the available evidence does not establish a specific circadian-mediated causal pathway for these outcomes, and established disease-specific determinants must remain central to their interpretation.

This distinction is particularly important for PUD. Helicobacter pylori infection and non-steroidal anti-inflammatory drug exposure remain major established etiological factors, while experimental evidence suggests that circadian, neuroendocrine, oxidative, and inflammatory processes may modify gastric mucosal defense [3,10,14,35,54,55,56]. H. pylori-associated inflammation can contribute to gastroduodenal injury and other long-term gastric complications [54,55,56]; however, current evidence does not demonstrate that occupational shift work causes H. pylori infection or materially modifies its pathogenicity in humans. Any interaction between shift-work-related physiological disturbances and H. pylori-associated disease should therefore be regarded as biologically plausible but insufficiently established.

For IBD, distinguishing mechanistic plausibility from occupational epidemiological evidence is particularly important. Experimental and translational studies provide substantial evidence that circadian clock disruption can influence epithelial barrier function, mucosal immunity, and host–microbiota interactions [16,17,18,19,20]. However, direct epidemiological evidence demonstrating an increased incidence of IBD among shift workers remains sparse. Notably, a recent systematic review and meta-analysis of gastrointestinal outcomes among nurses found no significant association between shift work and IBD [51]. Therefore, IBD should currently be regarded as a biologically plausible outcome within the proposed framework that requires prospective occupational validation rather than an established shift-work-associated gastrointestinal disease.

Beyond inflammatory disorders, chronic circadian disruption has also been implicated in colorectal carcinogenesis. The International Agency for Research on Cancer classified night-shift work involving circadian disruption as probably carcinogenic to humans (Group 2A), highlighting the potential long-term biological consequences of chronic occupational chronodisruption [26]. Experimental evidence indicates that disruption of molecular clock genes, persistent inflammation, oxidative stress, impaired immune surveillance, altered microbial metabolism, and epithelial barrier dysfunction may contribute to a pro-tumorigenic microenvironment [26,27,28,29,30,31,32,33,34]. However, the epidemiological relationship between shift work and colorectal neoplasia remains substantially less certain. An earlier meta-analysis reported an increased risk of CRC among night-shift workers and suggested an exposure–response relationship with longer night-shift work [51,57]. In contrast, subsequent cohort studies have produced inconsistent findings, and a later systematic review and meta-analysis of six studies examining shift work found no statistically significant association with colorectal neoplasms (RR = 1.06, 95% CI 0.95–1.17) [52]. More recent evidence synthesis has likewise failed to establish a consistent occupational association [51]. Thus, although experimental evidence provides biologically plausible pathways linking circadian disruption with colorectal carcinogenesis, current epidemiological evidence does not establish shift work as an independent causal risk factor for CRC. The CRC component of the proposed framework should therefore be interpreted as mechanistically plausible but epidemiologically unresolved.

Importantly, the absence of gastrointestinal disease in many shift workers may reflect not only differences in exposure intensity and individual susceptibility but also the activity of host compensatory and resilience mechanisms. Circadian and immune systems possess adaptive regulatory pathways that may partially counterbalance recurrent disruption, including anti-inflammatory and regulatory immune responses, epithelial repair and renewal, mucus and antimicrobial defenses, and restoration of neuroendocrine and circadian homeostasis during recovery periods. The intestinal microbiota may likewise exhibit ecological resilience, with microbial communities and metabolic functions partially recovering after transient perturbations when dietary, sleep–wake, and environmental conditions become more favorable. These buffering mechanisms may limit progression from transient physiological disturbance to persistent barrier, immune, or microbial dysfunction. Their effectiveness likely varies with exposure duration and intensity, recovery opportunities, chronotype, age, lifestyle, baseline microbiome characteristics, and pre-existing health status. However, the extent to which such compensatory mechanisms determine resilience to gastrointestinal disease in shift-working populations remains insufficiently characterized and represents an important area for longitudinal research.

Taken together, current epidemiological evidence supports an association between shift work and selected gastrointestinal outcomes, particularly IBS, while evidence for other conditions varies from limited or inconsistent occupational associations to predominantly experimental mechanistic plausibility. The complete sequence proposed in Figure 1—from occupational exposure through circadian, neuroendocrine, immune, epithelial, and microbial alterations to clinically manifest gastrointestinal disease—has not been demonstrated prospectively in humans. This distinction is essential when translating the mechanistic framework into occupational health implications and preventive strategies.

To facilitate comparison across gastrointestinal outcomes, Table 5 summarizes the current epidemiological evidence linking occupational shift work with major gastrointestinal disorders, while distinguishing relatively consistent human associations from limited or heterogeneous findings and disease relationships supported predominantly by mechanistic plausibility.

## 5. Occupational Health and Translational Implications

### 5.1. Risk Assessment and Identification of Potentially Susceptible Shift Workers

The heterogeneity of gastrointestinal outcomes among shift workers suggests that occupational exposure does not confer uniform risk. Individual susceptibility is likely to reflect interactions among schedule characteristics, sleep and meal timing, chronotype, lifestyle factors, psychosocial stress, and pre-existing gastrointestinal or metabolic conditions [1,6,23,24,25,26,27,28,29,30,31,32,33,34,58,59,60]. However, validated models for predicting gastrointestinal disease specifically among shift workers are currently lacking.

Work-schedule characteristics remain the most directly assessable component of occupational risk. Duration of shift-work exposure, frequency and number of consecutive night shifts, shift duration, rotation direction, recovery intervals, and permanent versus rotating schedules may influence circadian strain and health outcomes [1,6,23,24,25,26,27,28,29,30,31,32,33,34,58,59,60]. For some gastrointestinal outcomes, particularly IBS, recent prospective evidence also suggests exposure–response relationships with the duration and frequency of night-shift work [6]. Nevertheless, evidence is not sufficiently consistent across gastrointestinal diseases to define universal exposure thresholds for clinically meaningful risk.

Individual characteristics may further modify responses to non-standard work schedules. Chronotype can influence adaptation to night work, while age, sleep characteristics, diet, smoking, alcohol consumption, physical activity, psychological stress, obesity, metabolic disorders, and pre-existing gastrointestinal disease may contribute to interindividual variability [31,32,33,34,58,61,62,63]. Many of these factors are also independent determinants of gastrointestinal health, making it difficult to distinguish effect modification from confounding in observational studies.

At present, occupational gastrointestinal risk assessment should therefore remain primarily clinical and exposure-based rather than biomarker-driven. Assessment of work-schedule characteristics, gastrointestinal symptoms, sleep and meal timing, relevant lifestyle factors, medication use, and pre-existing gastrointestinal conditions may help identify workers who warrant closer clinical evaluation. Such assessment should not be interpreted as a validated gastrointestinal risk-prediction model.

Future prospective studies should determine whether combining detailed occupational exposure data with chronotype, sleep characteristics, dietary timing, and selected biological measures improves prediction beyond conventional clinical assessment. Until such models are externally validated, individualized risk stratification should be regarded as a research objective rather than an established component of occupational health surveillance.

### 5.2. Candidate Biomarkers and Priorities for Occupational Research

Biological markers may eventually help characterize the physiological effects of chronic shift-work exposure, but their potential role in gastrointestinal occupational health remains investigational. Conventional occupational health surveillance primarily relies on medical history, symptom assessment, and routine clinical examination, together with established disease-specific diagnostic approaches. At present, no biomarker or biomarker panel has been validated for screening, predicting, or diagnosing shift-work-associated gastrointestinal disease.

Given the multisystem nature of chronic circadian disruption, no single biomarker is likely to adequately capture the entire spectrum of biological alterations induced by shift work. Several biological domains nevertheless warrant investigation, including markers of circadian timing and neuroendocrine regulation, systemic inflammation and immune function, intestinal epithelial integrity, microbial composition, and microbial metabolism. Importantly, their current value should be considered primarily mechanistic and research-oriented rather than diagnostic.

Among circadian biomarkers, melatonin remains the most extensively investigated indicator of circadian integrity. Dim-light melatonin onset and nocturnal melatonin profiles can characterize circadian phase and alignment, while repeated cortisol measurements may provide complementary information regarding hypothalamic–pituitary–adrenal axis rhythmicity [10,24,25,35,61]. However, neither melatonin nor cortisol measurements have been validated as gastrointestinal risk biomarkers for occupational surveillance. Their interpretation is also sensitive to sampling time, light exposure, sleep timing, stress, medication use, and other environmental or behavioral factors.

Immune biomarkers constitute a second major component of biological surveillance. Researchers have investigated CRP and selected cytokines, including IL-6 and TNF-α, in relation to night work, sleep disruption, and circadian misalignment [11,12,13,14]. Immune-cell phenotyping may provide additional mechanistic information regarding leukocyte and lymphocyte responses to altered work schedules. However, these measures are nonspecific, may be influenced by numerous infectious, inflammatory, metabolic, and behavioral factors, and have not been validated for predicting gastrointestinal disease in shift workers. Their current relevance therefore lies primarily in characterizing biological responses to occupational circadian disruption rather than in routine clinical risk assessment.

Assessing intestinal barrier integrity is another potentially informative research approach. Studies of epithelial injury, microbial translocation, or intestinal permeability have investigated biomarkers such as intestinal fatty acid-binding protein (I-FABP), lipopolysaccharide-binding protein (LBP), circulating endotoxin-related measures, and zonulin [64,65,66,67,68]. However, these biomarkers reflect different biological processes and should not be interpreted as interchangeable measures of intestinal barrier dysfunction. I-FABP primarily reflects enterocyte injury rather than permeability itself, whereas LBP and circulating endotoxin-related measures provide indirect information on exposure to or translocation of bacterial products and may also be influenced by systemic inflammatory and metabolic conditions [64,65]. Zonulin has been widely used as a putative marker of intestinal permeability, but substantial methodological concerns regarding assay specificity and biological interpretation limit its reliability [64,66,67,68,69]. Consequently, no single circulating biomarker can currently be considered a definitive measure of intestinal permeability, and multimarker approaches should likewise be interpreted according to the specific biological process each component represents. Importantly, none of these measures has been validated for routine gastrointestinal surveillance in occupational shift-working populations. Their principal current value lies in prospective mechanistic studies examining whether circadian and immune alterations are accompanied by measurable changes in epithelial injury, permeability-related processes, or microbial translocation.

Growing interest has also focused on the diagnostic potential of the gut microbiome and its metabolites. Microbiome and metabolomic profiling represents an emerging research approach rather than an established diagnostic strategy. Functional assessment of microbial metabolism, including SCFAs, secondary bile acids, tryptophan-derived indoles, and related microbial products, may complement taxonomic profiling by providing information on host–microbiota metabolic interactions. However, diet, meal timing, geography, medication use, age, host characteristics, and analytical methodology strongly influence microbiome composition and function. Available occupational studies remain limited, and no reproducible microbial or metabolomic signature of gastrointestinal risk attributable specifically to shift work has yet been established.

Integrative approaches combining detailed occupational exposure assessment with circadian phenotyping, inflammatory markers, intestinal barrier measures, microbiome profiling, and metabolomics may be valuable in future research. Such studies could determine whether combinations of biological measures explain or predict gastrointestinal outcomes beyond conventional exposure and clinical variables. At present, however, the complexity, cost, lack of standardization, and absence of validated predictive thresholds preclude their use as routine occupational surveillance tools.

Although considerable progress has been achieved in identifying candidate biomarkers, several challenges remain before their routine implementation in occupational medicine. Most available evidence comes from small observational studies with heterogeneous populations, varying exposure definitions, and limited longitudinal follow-up. In addition, differences in biological sampling schedules, analytical platforms, and control of major confounders limit comparability across studies. Consequently, multicenter prospective cohorts incorporating standardized exposure definitions, repeated time-controlled biological sampling, prespecified gastrointestinal outcomes, and independent external validation will be required to determine the clinical relevance and predictive performance of candidate biomarkers.

Practical implementation also presents substantial challenges beyond analytical validation. Advanced circadian phenotyping, immune profiling, intestinal barrier-related assays, microbiome sequencing, and metabolomic analyses differ considerably in cost, technical complexity, availability, pre-analytical requirements, and interpretability, making most of them unsuitable for routine occupational health examinations at present. Their future translation would also require standardized sampling protocols, clinically interpretable thresholds, laboratory quality assurance, and evidence that they provide incremental predictive value beyond conventional occupational and clinical assessment. Moreover, integration of multidimensional biological information would require interdisciplinary collaboration among occupational physicians, gastroenterologists, chronobiologists or sleep specialists, laboratory medicine professionals, microbiologists, nutrition specialists, epidemiologists, and biostatistical or bioinformatics teams. These practical considerations further support maintaining clinical and exposure-based assessment as the current first-line approach while advanced biological profiling remains confined primarily to research settings.

The diversity of biological pathways involved in chronic circadian disruption highlights the need for an integrated rather than isolated biomarker strategy. Table 6 summarizes candidate biomarkers relevant to the proposed framework, their biological rationale, predominant evidence base, potential research applications, current implementation status, and the principal limitations preventing their use in routine occupational gastrointestinal surveillance.

Accordingly, candidate biomarkers should currently be regarded as tools for mechanistic investigation and hypothesis testing rather than as established instruments for screening or individualized occupational decision-making. Their future clinical utility will depend on demonstrating reproducibility, standardized measurement, incremental predictive value beyond conventional clinical and occupational exposure variables, and prospective associations with clearly defined gastrointestinal outcomes.

### 5.3. Potential Preventive Strategies and Current Evidence

The interventions discussed below should not be interpreted as established strategies for preventing gastrointestinal disease in shift workers. Their evidentiary basis varies substantially: some approaches are supported by occupational or controlled circadian studies, others derive primarily from evidence in general gastrointestinal or metabolic populations, whereas several remain mechanistically plausible but insufficiently tested in shift-working populations. Preventive strategies should therefore be considered according to both their biological rationale and the directness of evidence supporting gastrointestinal benefit in occupational settings.

The first, and arguably most effective, preventive intervention is optimizing work schedule design. Among the approaches discussed in this section, schedule design is supported most directly by occupational and circadian evidence. Forward-rotating schedules, limiting consecutive night shifts, ensuring adequate recovery intervals between shifts, avoiding excessively prolonged working hours, and reducing cumulative circadian strain have been proposed to improve adaptation and reduce physiological burden [32,33,34,40,41,42]. Workplace lighting strategies may also influence circadian adaptation, although their direct effects on gastrointestinal outcomes remain unclear. Thus, schedule optimization represents a reasonable occupational health measure, but gastrointestinal disease prevention should not be inferred from circadian benefits alone.

Lifestyle interventions constitute a second essential component of prevention. Meal timing, diet quality, sleep optimization, hydration, and physical activity matter because shift work often disrupts several of these behaviors at once. Chrononutrition, including more regular food intake and avoiding large meals during the biological night, has a strong circadian and metabolic rationale, while balanced dietary patterns and adequate fiber intake are supported by broader gastrointestinal health evidence. However, direct intervention studies demonstrating that these strategies prevent gastrointestinal disease specifically in shift workers remain limited. Accordingly, they should be regarded as supportive health measures with plausible gastrointestinal benefits rather than as validated shift-work-specific preventive interventions.

Stress management, adequate sleep, regular physical activity, and maintenance of healthy body weight may also contribute to more favorable inflammatory and metabolic profiles. However, evidence that these interventions prevent shift-work-associated gastrointestinal disease through specific immunological mechanisms is insufficient. Similarly, immune biomarkers should not currently be used to select workers for intensified preventive interventions, because no biomarker-guided strategy has been prospectively validated for this purpose.

Interventions directed toward intestinal barrier function and the gut microbiota require even greater caution. Dietary fiber and overall diet quality have established relevance to gastrointestinal health, whereas prebiotics, probiotics, synbiotics, postbiotics, and other microbiota-directed approaches show context-dependent effects in general gastrointestinal populations and experimental models. Evidence demonstrating prevention of gastrointestinal disease through these interventions specifically in shift workers remains insufficient. Barrier-targeted and microbiota-directed approaches should therefore currently be considered experimental or investigational in the occupational context rather than recommended preventive strategies.

Melatonin and other chronobiotic approaches may also be relevant to circadian adaptation, but evidence supporting their routine use specifically to prevent gastrointestinal disease in shift workers is insufficient. Their potential gastrointestinal effects should therefore be distinguished from evidence concerning sleep or circadian phase adjustment, and dedicated occupational intervention trials are required before gastrointestinal preventive recommendations can be made.

Importantly, the current evidence does not support personalized preventive strategies based on chronotype, inflammatory profiles, intestinal permeability markers, or microbiome signatures. Individual characteristics such as chronotype, sleep quality, pre-existing gastrointestinal disease, metabolic status, and cumulative occupational exposure may reasonably inform clinical assessment, but biomarker-guided or molecularly individualized prevention remains investigational.

Despite encouraging mechanistic evidence, several important challenges remain. Most preventive interventions have been evaluated in relatively small studies, frequently targeting isolated components of circadian disruption rather than gastrointestinal outcomes in well-characterized occupational populations. Future randomized or prospective intervention studies should incorporate standardized shift-work exposure assessment, appropriate circadian and behavioral measures, and predefined gastrointestinal outcomes. Mechanistic biomarkers, immune phenotyping, and microbiome or metabolomic profiling may be included as research endpoints, but they should not replace clinically relevant gastrointestinal outcomes.

Given the heterogeneous evidence supporting these approaches, Table 7 summarizes the principal potential preventive strategies, distinguishing evidence from occupational or controlled circadian studies from evidence from general gastrointestinal populations and from interventions that remain predominantly experimental or insufficiently tested in shift workers.

While these preventive approaches provide a practical framework for reducing occupational circadian strain, their translation into gastrointestinal risk reduction requires stronger prospective evidence and consideration of the broader occupational context. The following section therefore examines the implications of the current evidence for occupational health practice and identifies priorities for future implementation and research.

Overall, the translational implications of the current evidence are more robust for reducing occupational circadian strain and addressing modifiable behavioral factors than for biomarker-guided risk stratification or mechanism-specific gastrointestinal prevention. Future occupational strategies should therefore prioritize feasible, evidence-supported measures while prospective studies determine whether biological markers or targeted interventions provide additional clinical benefit. This evidence-proportionate approach avoids premature translation of mechanistic findings while preserving a clear pathway for future occupational gastrointestinal research.

## 6. Future Perspectives and Research Priorities

Despite increasing recognition of the gastrointestinal consequences of chronic shift work, several important gaps continue to limit translation of mechanistic findings into occupational health practice. Current evidence comes predominantly from cross-sectional or observational studies, often involving heterogeneous occupational populations and inconsistent definitions of shift work. Future research should therefore prioritize large prospective longitudinal cohorts that standardize shift-schedule characterization, including night-shift frequency, rotation direction, consecutive night duties, shift duration, recovery intervals, cumulative years of exposure, and individual chronotype. Whenever possible, studies should incorporate appropriate fixed-day comparison groups and repeated exposure assessment to account for changes in work schedules over time. Such harmonization would improve cross-study comparisons and enable more robust evaluation of exposure–response relationships.

A particularly important priority is to test, rather than assume, the proposed sequence linking occupational exposure, circadian misalignment, neuroendocrine alterations, immune dysregulation, intestinal barrier dysfunction, microbial changes, and gastrointestinal outcomes. Few human occupational studies have simultaneously assessed multiple components of this pathway, and evidence that one stage prospectively mediates the next remains limited. Longitudinal designs incorporating temporally ordered measurements and appropriate mediation or causal-inference approaches will be required to determine which proposed transitions occur in human shift workers and which remain primarily mechanistic hypotheses.

A second priority is shifting from isolated biomarker measurements to integrated longitudinal biological profiling. Most available studies have evaluated circadian, inflammatory, metabolic, or microbial alterations separately, despite the interconnected nature of these pathways. Future mechanistic studies could combine objective measures of circadian alignment, including melatonin and cortisol rhythmicity, with immune phenotyping, inflammatory mediators, intestinal barrier biomarkers, microbiome profiling, and microbial metabolomics. Repeated sampling across different phases of the shift cycle would be particularly valuable, as single time-point measurements may miss biologically meaningful circadian alterations. Importantly, such measures should initially be considered research endpoints rather than validated tools for occupational screening or gastrointestinal risk prediction.

The gut microbiome and intestinal barrier represent important but insufficiently characterized research areas. Future investigations should move beyond descriptive taxonomic comparisons toward functional assessment using metagenomics, metabolomics, and quantification of microbial-derived metabolites such as SCFA, secondary bile acids, and tryptophan derivatives. Simultaneous assessment of complementary markers reflecting epithelial injury, permeability-related processes, and microbial translocation may help determine whether microbiome alterations precede barrier dysfunction, result from it, or participate in a bidirectional amplification loop during chronic occupational chronodisruption. These studies should carefully control for major determinants of microbiome composition, particularly diet and meal timing, medication use, age, metabolic status, geography, and other lifestyle factors.

Another important research direction concerns interactions between shift work and established gastrointestinal risk factors. In particular, the relationship between circadian disruption and *H. pylori* infection warrants further investigation. Rather than considering shift work as a cause of infection, future studies should determine whether chronic circadian and neuroimmune disruption modifies host responses to *H. pylori*, bacterial persistence, gastric mucosal inflammation, epithelial repair, or susceptibility to peptic ulcer complications. Similar interaction-based approaches should evaluate dietary patterns, obesity, smoking, alcohol consumption, medication exposure, psychological stress, and pre-existing gastrointestinal disease as potential modifiers of individual susceptibility.

Future longitudinal studies should also investigate determinants of biological resilience, including the capacity for circadian re-entrainment, resolution of inflammatory responses, epithelial repair, and recovery of microbial community structure and function after repeated shift-work exposure.

Importantly, observational associations must increasingly be complemented by interventional research. Randomized or carefully controlled prospective studies should determine whether optimization of shift rotation, appropriately timed light exposure, chrononutrition, improved sleep scheduling, stress-management approaches, physical activity, or microbiota-directed interventions can improve gastrointestinal symptoms or reduce the incidence of clinically defined gastrointestinal outcomes in shift workers. Because the evidence supporting these interventions differs substantially, trial priorities should reflect the strength of their existing occupational, circadian, and gastrointestinal evidence rather than assuming equivalent preventive efficacy. Mechanistic endpoints should accompany clinical outcomes to establish whether improvement is mediated through restoration of circadian alignment, attenuation of inflammation, preservation of epithelial barrier integrity, or normalization of microbial function.

Advanced multi-omics and computational approaches may eventually help integrate complex occupational and biological datasets, but their role should currently be regarded as exploratory. Combining occupational exposure characteristics with circadian, immunological, epithelial, microbial, metabolic, and clinical data could help identify reproducible biological patterns associated with differential gastrointestinal susceptibility. Machine-learning approaches may facilitate such analyses, particularly in high-dimensional longitudinal datasets; however, any resulting predictive models would require independent external validation, comparison with simpler clinical and exposure-based models, assessment of calibration and incremental predictive value, and demonstration of clinical utility before occupational application. Ethical considerations concerning biological monitoring, privacy, employment-related discrimination, data governance, and the appropriate use of individual risk estimates must also be addressed before such approaches can move beyond research settings.

Ultimately, the principal research challenge is to determine which components of the proposed framework are reproducibly observed in human shift workers, whether they temporally and causally contribute to specific gastrointestinal outcomes, and whether modifying relevant occupational or behavioral exposures improves clinically meaningful endpoints. Particular attention should be given to disease-specific evidence, because the strength of current epidemiological associations differs substantially between IBS and other outcomes such as functional dyspepsia, IBD, and CRC. Adequately powered prospective cohorts and intervention studies are essential before mechanistic findings, candidate biomarkers, or targeted interventions can be incorporated into evidence-based occupational gastrointestinal surveillance and prevention.

## 7. Conclusions

Chronic shift work represents an important occupational exposure associated with circadian misalignment and an increased burden of selected gastrointestinal outcomes. However, the strength of epidemiological evidence differs substantially across gastrointestinal disorders, with the most consistent evidence currently concerning IBS, whereas associations with functional dyspepsia, IBD, CRC, and other gastrointestinal conditions remain limited, inconsistent, or unresolved. This review proposes an integrative framework linking occupational shift exposure with circadian disruption, neuroendocrine alterations, immune dysregulation, intestinal barrier dysfunction, and changes in host–microbiota interactions. Importantly, this framework should be regarded as hypothesis-generating rather than as an established linear causal cascade in human shift workers. Available human evidence supports associations between shift work, circadian disruption, and selected gastrointestinal outcomes, while experimental studies provide mechanistic plausibility for interconnected neuroendocrine, immune, epithelial, and microbial pathways. Direct longitudinal evidence demonstrating the complete sequence and its causal contribution to gastrointestinal disease remains limited. Future research should therefore prioritize prospective occupational cohorts and intervention studies using standardized shift-work definitions, temporally repeated biological measurements, and clinically relevant gastrointestinal outcomes. Candidate biomarkers and mechanism-based interventions remain investigational and require prospective validation before routine occupational application.

## Figures and Tables

**Table 1:** Evidence hierarchy across the proposed mechanistic cascade linking shift work to gastrointestinal disease.

Proposed Transition	Predominant Evidence Base	Evidence Category	Interpretation
Shift work → circadian disruption	Human occupational and controlled human circadian studies	I–II	Strong evidence that night/rotating work disrupts sleep–wake timing, melatonin rhythms, and circadian alignment
Circadian disruption → neuroendocrine alterations	Human shift-worker and controlled circadian studies	I–II	Melatonin suppression and altered cortisol rhythmicity are documented, although magnitude varies with schedule and individual adaptation
Neuroendocrine alterations → immune dysregulation	Human observational studies complemented by experimental studies	II–III	Circadian and neuroendocrine perturbations are associated with altered inflammatory signaling and immune rhythmicity, but longitudinal mediation in workers remains incompletely demonstrated
Immune dysregulation → intestinal barrier dysfunction	Predominantly mechanistic human and experimental/animal evidence	III	Strong biological plausibility and experimental support, but limited direct demonstration in occupational cohorts
Barrier dysfunction ↔ gut dysbiosis/metabolic remodeling	Experimental, animal, and non-occupational human microbiome studies	III	Bidirectional interactions are well supported biologically, whereas shift-worker-specific longitudinal evidence remains limited
Integrated pathway → gastrointestinal disease	Occupational epidemiology supports disease associations; complete mechanistic mediation remains unproven	II–IV	Shift work is associated with several gastrointestinal outcomes, but the complete sequential cascade has not been demonstrated prospectively in humans

**Table 2:** Circadian regulation of immune-cell populations and their alterations in chronic shift workers.

Immune Component	Physiological Circadian Role	Alterations Associated with Chronic Shift Work	Potential Gastrointestinal Consequences
Neutrophils	Circadian trafficking, microbial defense, early inflammatory response	Altered migration, prolonged activation, increased inflammatory responsiveness	Enhanced epithelial injury and oxidative stress
Monocytes/Macrophages	Phagocytosis, tissue repair, maintenance of intestinal immune tolerance	Increased pro-inflammatory phenotype, impaired inflammatory resolution	Persistent mucosal inflammation and impaired barrier repair
Dendritic cells	Circadian antigen presentation and T-cell priming	Dysregulated antigen presentation and immune activation	Loss of immune tolerance toward luminal antigens
Natural killer cells	Circadian cytotoxic activity and immune surveillance	Reduced cytotoxic function and altered cytokine secretion	Impaired immune surveillance and chronic inflammatory activation
Innate lymphoid cells	Maintenance of epithelial integrity and mucosal defense	Disturbed epithelial surveillance and cytokine production	Increased susceptibility to epithelial barrier dysfunction
CD4^+^ T lymphocytes	Coordination of adaptive immune responses	Altered activation and cytokine production	Exaggerated mucosal inflammatory responses
Regulatory T cells (Tregs)	Maintenance of immune tolerance	Reduced regulatory activity and impaired immune suppression	Loss of intestinal immune tolerance
T helper 17 cells	Mucosal host defense and epithelial protection	Excessive pro-inflammatory activation	Chronic intestinal inflammation and epithelial injury
B lymphocytes	Antibody production and mucosal immune protection	Altered humoral immune responses	Impaired mucosal immune homeostasis

Summary of the principal innate and adaptive immune-cell populations regulated by circadian rhythms, their physiological functions, alterations associated with chronic occupational shift work, and their potential contribution to gastrointestinal dysfunction. Collectively, these immune alterations provide the mechanistic link between neuroendocrine dysregulation and intestinal barrier impairment within the proposed shift work–gastrointestinal disease cascade.

**Table 3:** Structural and functional components of the intestinal barrier affected by chronic shift work.

Barrier Component	Physiological Function	Effect of Chronic Shift Work	Potential Gastrointestinal Consequence
Mucus layer (Goblet cells)	Physical separation of microbiota from epithelium	Reduced mucus secretion and altered mucus composition	Increased microbial contact with epithelium
Intestinal epithelial cells	Selective permeability and nutrient absorption	Impaired regeneration and epithelial integrity	Increased intestinal permeability
Tight-junction proteins (Claudins, Occludin)	Regulation of paracellular permeability	Tight-junction disruption mediated by inflammatory cytokines	“Leaky gut” and microbial translocation
Paneth cells	Antimicrobial peptide secretion	Altered antimicrobial peptide production	Reduced mucosal defense
Resident immune cells	Immune surveillance and tolerance	Persistent inflammatory activation	Loss of immune homeostasis
Pattern-recognition receptors	Detection of microbial products	Chronic overstimulation by translocated microbial components	Sustained NF-κB and inflammasome activation

Principal structural and immunological components of the intestinal barrier and the mechanisms through which chronic circadian disruption associated with shift work impairs barrier integrity. Alterations affecting epithelial cells, mucus production, tight-junction proteins, antimicrobial defenses, and mucosal immune surveillance collectively increase intestinal permeability and facilitate the transition toward gut microbial dysbiosis and gastrointestinal disease.

**Table 4:** Principal gut microbial metabolites involved in circadian gastrointestinal homeostasis and their alterations during chronic shift work.

Microbial Metabolite	Physiological Function	Alteration Associated with Chronic Shift Work	Potential Gastrointestinal Consequence
Butyrate	Energy source for colonocytes; maintenance of tight junctions	Reduced production	Barrier dysfunction and increased permeability
Acetate	Regulation of epithelial metabolism and immune responses	Altered microbial production	Impaired mucosal homeostasis
Propionate	Immune regulation and metabolic signaling	Decreased availability	Enhanced inflammatory activation
Secondary bile acids	FXR/TGR5 signalling; epithelial regeneration	Altered bile acid metabolism	Disturbed epithelial repair and microbial ecology
Indole derivatives (Tryptophan metabolites)	AhR activation; IL-22 production; epithelial protection	Reduced microbial biotransformation	Loss of mucosal immune tolerance
Lipopolysaccharide	Component of Gram-negative bacteria	Increased systemic translocation	Persistent TLR-mediated inflammation

Major microbial-derived metabolites involved in maintaining intestinal barrier integrity, immune regulation, and gastrointestinal homeostasis. Chronic circadian disruption associated with shift work alters microbial metabolic activity, thereby amplifying epithelial dysfunction, immune activation, and chronic inflammation through multiple host–microbiota signaling pathways.

**Table 5:** Gastrointestinal disorders associated with chronic shift work and the principal biological mechanisms involved.

Gastrointestinal Disorder	Principal Mechanisms	Strength of Current Evidence	Key Occupational Implications
Irritable bowel syndrome	Circadian disruption, gut–brain axis dysfunction, barrier impairment, dysbiosis	Relatively strong (multiple meta-analyses and cohort studies)	Early recognition and risk assessment in long-term shift workers
Functional dyspepsia	Altered gastric motility, neuroendocrine dysregulation, low-grade inflammation	Limited–moderate	Symptom monitoring and chrononutrition strategies
Gastroesophageal reflux disease	Altered gastric emptying, autonomic dysfunction, irregular meal timing	Moderate	Meal scheduling and sleep hygiene interventions
Peptic ulcer disease	Reduced mucosal protection, melatonin deficiency, oxidative stress, chronic inflammation	Moderate	Assessment of additional risk factors (*H. pylori*, NSAID use)
Inflammatory bowel disease	Immune dysregulation, epithelial barrier dysfunction, altered microbiota	Limited; no consistent occupational association	Monitoring susceptible individuals; further prospective studies needed
Colorectal cancer	Chronic inflammation, oxidative stress, immune surveillance impairment, molecular clock disruption	Inconsistent/weak epidemiological evidence	Long-term surveillance and cumulative exposure assessment

Evidence descriptors reflect the consistency, directness, and population specificity of the available occupational epidemiological data and should not be interpreted as a formal evidence-grading system.

**Table 6:** Candidate biomarkers for investigating the biological effects of shift work and their current evidence and limitations for occupational gastrointestinal research.

Candidate Domain	Candidate Measures	Biological Rationale	Predominant Evidence Base	Potential Research Application	Current Implementation Status	Current Limitations
Circadian timing	Melatonin/DLMO	Circadian phase/alignment	Human circadian + occupational	Circadian phenotyping	Specialized/research assessment	Timing- and light-sensitive sampling; logistical requirements; no validated GI predictive thresholds
HPA axis	Repeated cortisol profiles	Neuroendocrine rhythmicity	Human observational/circadian	Physiological characterization	Specialized/research assessment	Stress- and timing-sensitive; influenced by sleep, medications, and other confounders; no GI predictive validation
Inflammation	CRP, IL-6, TNF-α	Systemic inflammatory state	Human observational	Mechanistic/longitudinal research	Technically accessible, but not validated for GI risk stratification	Nonspecific; influenced by infection, metabolic disease, obesity, lifestyle, and sampling conditions
Immune phenotype	Leukocyte subsets, NK/T-cell parameters	Circadian immune regulation	Mixed human/experimental	Mechanistic research	Investigational	Technically complex; time-dependent variation; limited standardization; no validated predictive thresholds
Intestinal barrier-related processes	I-FABP, LBP, endotoxin-related measures; zonulin with caution	Different aspects of enterocyte injury, microbial translocation, or permeability-related processes	Mainly experimental/general GI	Mechanistic studies	Investigational	Markers are not interchangeable; indirect or process-specific measures; assay and specificity limitations; no validated occupational thresholds
Microbiome	Metagenomic profiles	Host–microbiota interactions	Experimental + limited human	Discovery research	Investigational	High interindividual variability; major dietary, medication, geographic, and methodological influences; no validated occupational GI-risk signature
Metabolomics	SCFAs, bile acids, indoles	Functional microbial activity	Mainly experimental/general GI	Mechanistic/discovery research	Investigational	Analytical and temporal variability; limited standardization; cost; no validated occupational thresholds

Current implementation status reflects the level of validation for occupational gastrointestinal assessment rather than the assay’s technical availability. “Specialized/research assessment” indicates measures that can be performed in specialized settings but are not validated for gastrointestinal risk prediction in shift workers. “Technically accessible, but not validated for GI risk stratification” indicates routinely measurable biomarkers whose availability should not be interpreted as evidence of predictive utility for shift-work-associated gastrointestinal outcomes. “Investigational” indicates measures for which current evidence is insufficient to support routine occupational application. These categories describe implementation readiness and do not constitute a formal evidence-grading system.

**Table 7:** Current evidence supporting potential preventive strategies for gastrointestinal health in shift-working populations.

Potential Intervention	Occupational/Circadian Evidence	General Gastrointestinal Evidence	Direct Evidence in Shift Workers for GI Outcomes	Current Interpretation
Work-schedule optimization	Human occupational and circadian studies	Indirect evidence relevant to GI health	Limited direct studies	Supported for circadian/occupational health; GI-specific preventive effect not established
Workplace light management	Human occupational and controlled circadian studies	Limited GI-specific evidence	Insufficient direct evidence	Supports circadian adaptation; GI benefit unproven
Sleep optimization	Human occupational and sleep/circadian studies	Associations between sleep characteristics and GI health reported in human studies	Limited direct studies	Reasonable supportive measure; GI-specific efficacy uncertain
Meal timing/chrononutrition	Controlled human circadian/metabolic studies; limited occupational studies	Human and experimental evidence linking meal timing with metabolic and GI-related physiology	Limited direct studies	Biologically plausible; occupational GI trials needed
Diet quality and fiber	Limited shift-worker-specific evidence	Established nutritional and GI-health evidence in general populations	Limited direct studies	General GI health measure; shift-specific preventive effect uncertain
Physical activity/weight management	General occupational and health evidence	Human evidence linking physical activity and weight status with GI and metabolic health	Limited direct studies	General health measure; specific GI prevention unproven
Stress management	Occupational and general human evidence	Human evidence relevant particularly to disorders of gut–brain interaction	Limited direct studies	Potentially relevant; shift-worker GI efficacy insufficiently tested
Probiotics/prebiotics/postbiotics	Very limited occupational evidence	Context-dependent clinical evidence in selected GI conditions	Insufficient direct evidence	Investigational for shift-work-related GI prevention
Barrier-targeted approaches	Predominantly experimental evidence	Experimental and limited clinical evidence concerning selected barrier-related interventions	No established direct evidence	Investigational
Melatonin/chronobiotic approaches	Human circadian evidence; limited GI-specific occupational evidence	Clinical evidence is indication- and outcome-dependent	Insufficient direct evidence	GI preventive use remains investigational

Evidence is characterized according to its predominant source, directness, and population specificity rather than by a formal grading system. Descriptors indicating limited or insufficient evidence refer to the availability of direct studies relevant to the specified population and outcome. Evidence supporting circadian, metabolic, or general gastrointestinal effects should not be interpreted as evidence of gastrointestinal preventive efficacy in shift-working populations.

## Data Availability

The data used to support the findings of this study are available from the corresponding author upon reasonable request.
